# The mechanistic insights into the application of retinoids and possible adjunct therapeutic treatment to androgenic alopecia

**DOI:** 10.1097/MS9.0000000000004153

**Published:** 2025-10-16

**Authors:** Hamza Irfan, Ahmad Asad Raza, Hira Amran Chowdhry, Muhammad Bin Mobin, Abedin Samadi

**Affiliations:** a Department of Medicine, Rashid Latif Medical College, Lahore, Pakistan; b Department of Medicine, Jinnah Sindh Medical University, Karachi, Pakistan; c Department of Medicine, Kabul University of Medical Sciences Abu Ali Sina, Kabul, Afghanistan

**Keywords:** androgenetic alopecia, dihydrotestosterone (DHT), hair follicle miniaturization, hair regeneration therapy, retinoids

## Abstract

Androgenetic alopecia (AGA) is a common, nonscarring hair loss condition affecting both men and women, often leading to psychological distress and reduced self-esteem. It involves the gradual miniaturization of hair follicles and a shortened anagen phase, primarily influenced by genetic predisposition and androgenic activity, especially dihydrotestosterone. The paradoxical role of androgens – promoting hair growth in some areas while suppressing it on the scalp – has been key in understanding AGA’s pathogenesis. Altered androgen receptor signaling in dermal papilla cells contributes significantly to follicular regression.

Current treatments offer limited success, often failing to reverse the progression of hair loss. However, recent research has turned attention toward retinoids – vitamin A derivatives known for their role in cell proliferation and differentiation. These compounds act via nuclear receptors to modulate gene expression and may promote hair growth by extending the anagen phase and stimulating follicular activity. Early studies suggest that topical retinoids, especially in combination with existing therapies, can improve treatment outcomes.

This evolving insight into retinoid biology and its interaction with hair follicle pathways offers a promising direction for more effective, mechanism-based treatments of AGA, potentially advancing long-term management and hair restoration options.

## Introduction

Androgenetic alopecia (AGA) is a widespread condition that is a significant cause of concern in people across the globe, affecting both genders^[1]^. AGA refers to progressive hair loss of the scalp, which is nonscarring in nature and consists of characteristic miniaturization of the hair follicles^[2]^. Ultimately, it results in hair follicles that no longer serve their purpose, rendering them nonfunctional^[1]^.

Factors that have been shown to significantly contribute to the development of AGA are genetics and androgens^[1]^. Other lifestyle factors such as smoking, stress, and poor diet have also been shown to contribute to the clinical presentation of AGA. AGA occurring in men and women is referred to as male AGA (MAGA) and female AGA (FAGA), respectively^[1]^. According to Alessandrini *et al*, AGA is prevalent in 80% of men and 50% of women and presents differently in both genders^[3]^. Male pattern baldness presents primarily at the fronto-temporal region and the vertex, while in women, the presentation may vary into three distinct types: diffuse thinning at the crown with sparing at the frontotemporal region, thinning and widening of the central part, and thinning associated with bitemporal recession^[4]^.

**HIGHLIGHTS**

- Retinoids show promise in treating AGA by extending the anagen phase.
- DHT and genetic factors are key in hair follicle miniaturization in AGA.
- Current drugs like finasteride and dutasteride have efficacy and side effects.
- Combined retinoid–minoxidil therapies enhance hair regrowth outcomes.
- Future research aims to optimize retinoid formulations and delivery methods.

Studies show that AGA has an adverse psychological effect on an individual’s perception of themselves. It is reported that women suffer more from negative body perceptions and low self-esteem in contrast to their counterparts^[5]^. For either gender, the process of AGA can be taxing and leads to unnecessary stress for individuals who suffer from the disease.

Although hair growth and regulation a complex mechanism that is not entirely understood, literature has shown that the use of retinoids helps in hair growth by targeting the regression of the hair follicles^[6]^. Retinoids refer to molecules derived from or that are structurally similar to Vitamin A and bind to retinoic acid (RA) receptors, resulting in activation and transcription of retinoic acid. RA is known to enhance cell proliferation and differentiation^[7]^. The use of topical retinoids has been shown to improve the clinical presentation of alopecia^[8]^. Although retinoids are well-established in dermatological therapy^[7,8]^, their role in AGA has received comparatively little mechanistic and clinical attention^[9]^. Addressing this gap is critical to determine whether retinoids represent merely an adjunctive agent or a potential stand-alone therapy.

This paper aims to understand the underlying pathological mechanisms that may give rise to AGA, available treatment options alongside their benefits and limitations, and the need for novel therapeutic alternatives that may be more effective in curing AGA. This paper will progress to evaluate the mechanism of action of Retinoids, their use as a possible treatment modality in multiple trials, and the possibility of Retinoids as a novel alternative to the treatment of AGA.

### Methodology

This study was conducted as a narrative review exploring the potential role of retinoids in treating AGA. Relevant articles were identified through a comprehensive search of PubMed, MEDLINE, Embase, Cochrane Library, and Scopus from inception to the present, using keywords such as “androgenetic alopecia,” “retinoids,” “retinoic acid,” and “tretinoin.” Studies were included if they investigated the use of topical or systemic retinoids in adults aged 18 years or older diagnosed clinically with AGA, and reported outcomes such as hair count, hair thickness, hair growth assessments, or adverse effects. Excluded were animal studies, in vitro research, case reports with fewer than 10 participants, studies focusing on alopecia types other than AGA, and articles without available full text or not published in English. Data extracted included study design, sample size, participant characteristics, type and formulation of retinoid, treatment duration, and outcomes. The quality of included studies was assessed using appropriate risk-of-bias tools, and findings were synthesized narratively due to anticipated heterogeneity in study designs and outcomes. The writing and data synthesis process adhered to the TITAN Guidelines for Transparent Integration of AI Tools in Academic Writing to ensure clarity, integrity, and responsible AI use.^[10]^

## Androgenetic alopecia: current understanding

In AGA, the most interesting part of androgen effects is the “androgen paradox,” which occurs. Kidangazhiathmana *et al* showed that under this paradoxical cycle, androgens promote growth in dependent areas such as axilla, pubis, and beard. However, in susceptible individuals, the effect is miniaturization of hair and a reduction in growth during the anagen stage (stage in which hair growth occurs)^[11]^.

Androgens such as testosterone are activated and converted to dihydrotestosterone (DHT) by 5α-reductase, which itself has two types, and recently studies have shown that 5aR2 is predominant in dermal papilla cells (DPC) from AGA and beard, and 5aR1 is seen in all scalp sites as expressed by Kidangazhiathmana^[11]^. Additionally, androgen receptors (AR) were found to be more in DPCs from the balding scalp than from nonbalding. AGA is considered to be an autosomal dominant inheritance trait and strongly associated with the X chromosome, with implication of the AR gene and ectodysplasin A2 receptor (AR/EDA2R locus in X111-q12), and finally supports evidence of the WNT pathway, as was seen in studies conducted by Heilmann *et al*^[12]^.

The pathogenesis of AGA revolves around two components: first, the hair follicle miniaturization, and second, the changes to the hair cycle and decrease in the anagen stage. Although the actual mechanism of hair follicle miniaturization is not fully established, the basic pathology shows that terminal hair is converted to Vellus hair. Hair follicles, in essence, have two parts: the ectodermal part with invagination of the epidermis and the hair bulb, which contains the hair shaft, and the mesenchymal part, which is the dermal papilla^[12]^.

The leading processes believed to contribute toward AGA are stated by Whiting as an abrupt reduction in cells of the hair papilla.^[2]^ On the other hand, the alternative process proposed by Gurrera and Rabora stated that accelerated mitotic processes in the hair can lead to reduced time for the necessary differentiation, increased telogen shedding, and eventually a prolonged lag phase.^[13]^

In the hair growth cycle, there are four identified phases of growth (anagen), involution (catagen), resting (telogen), and shedding (exogen)^[14]^. Certain growth factors, such as the insulin-like growth factor 1, hepatocyte growth factor, keratinocyte growth factor, and vascular endothelial growth factor, promote the anagen phase of the hair cycle. Similarly, the onset of the catagen phase is promoted by transforming growth factor beta, interleukin 1-alpha, and tumor necrosis factor-alpha^[12]^. In AGA, the anagen to telogen ratio is changed, and with the successive shortening of hair cycle, the hair shaft length is reduced, eventually ensuing an empty follicular pore wherein the hair fails to acquire even the minimum length needed to reach the skin surface. This is a prolonged phase of telogen, identified as the substage kenogen, and lasts longer in AGA, resulting in higher percentage of empty hair follicles^[15]^.

The process of miniaturization does not affect all of the hair follicles, which are in a follicular unit (FU) during AGA, but rather the secondary follicles are affected initially, which leads to thinning of hair, after which the primary follicles are affected. Miniaturization occurs between anagen cycles, and it is believed that there is cell movement between the dermal papilla and the dermal sheath, and the disruption of this movement in AGA causes cellular loss from the sheath and then the papilla, and this ultimately leads to miniaturization^[16]^.

### Pathology of androgenetic alopecia

In AGA the reduction in size of the dermal papilla is believed to affect the size of the hair bulb and shaft produced and although no exact theory proves why this change occurs, some mechanisms are thought of to contribute to this reduction which as apoptotic cell death, decreased proliferation of keratinocytes, displacement with subsequent loss of cell adhesion inducing fibroblast loss or cellular migration from pappila to sheath associated with outer root sheath of hair follicle^[17]^.

Early on in AGA, there is focal basophilic degeneration of the connective tissue of anagen and progressive miniaturization. As the disease advances, the vellus follicles also disappear, and decreased hair diameter with an increase in telogen hairs is appreciated. There is an increase in pseudo-vellus hair, a decrease in terminal hair, and the presence of residual angiofibrotic tracts, and ultimately, the terminal vellus ratio becomes <4:1^[11]^.

### Current treatment options for AGA

In the treatment of AGA, the use of 5α-reductase inhibitors (5ARI) has shown successful outcomes for patients who were affected by the disease. Finasteride and dutasteride are 5ARI, which have shown promising results as they inhibit the conversion of testosterone to DHT. In a study conducted, 1553 men who were treated with finasteride showed an improvement in hair counts. The treatment was able to slow the progression of hair loss in men with AGA^[18]^. Dutasteride, another 5ARI, has been used previously and shown positive effects on AGA, and countries such as Taiwan, Japan, and South Korea have approved its use for AGA^[19]^. Over a treatment course of 6 months, researchers found that dutasteride had a higher efficacy on hair growth. In a comparison of finasteride and dutasteride, the latter is more potent as it is a 5ARI type I and II inhibitor and leads to a 90% decrease in DHT levels^[20]^.

Although both finasteride and dutasteride have had positive effects, and the efficacy of both drugs has shown that they are helpful in the treatment of AGA in men, the efficacy of dutasteride was seen to be more than finasteride. In a study conducted with 917 men, Gubelin Harcha *et al* showed the effect of dutasteride compared to finasteride compared to a placebo on AGA in men. The results showed that not only was utasteride more effective at increasing hair count at week 24 compared to its alternatives and had an overall better dose-dependent effect^[21]^. Additionally, two meta-analyses demonstrated that the efficacy of oral dutasteride in treating male AGA was significant as compared to oral finasteride. In the meta-analyses, overall 23 studies were included, of which there were 21 randomized trials and 2 single-group observational studies, and showed that after 24 weeks of daily 0.5 mg oral dutasteride, the outcome on hair growth was much better. In comparison to finasteride, the difference was 7.1 hairs/cm^2^, showing that dutasteride was more effective^[22]^.

A common complication of finasteride use that was seen in patients was erectile dysfunction and decreased libido. Studies showed that patients who had been treated with finasteride reported erectile dysfunction in up to 15.8% and decreased libido in 10% and ejaculatory dysfunction in about 5.7%^[23]^. However, it is noteworthy that finasteride is often used as a drug of choice for BPH, and this itself contributes to erectile dysfunction as seen in a systematic review by Kiguradze *et al*^[16]^. In the systematic review, 10 studies supported evidence of sexual dysfunction with to use of finasteride, while 5 did not support this evidence^[17]^. On the other hand, there were no studies that reported significant dysfunction in sexual activity with the use of dutasteride^[24]^.

### Side effects of current treatments

The effect of these treatment on infertility in men was also recorded in regards of their sperm count, sperm concentration and sperm motility and it was shown that Finasteride decreased these values in male patients using over 5 mg daily doses of finasteride; however, the value for sperm volume and concentration did return after discontinuation, the sperm count in patients did not recover as much^[25]^. Similar studies conducted with the effect of dutasteride showed that for treatment using 0.5 mg dutasteride, there was a significant decrease in sperm count in the initial 26 weeks of treatment but not much different in the total 52 weeks nor in the 24 weeks follow-up phase which was conducted and although the studies conducted showed a negative effect of dutasteride on the sperm count, motility and volume, the overall impact was unclear and it may be that fertility is not altogether impacted and some patients may be more sensitive to the effect of dutasteride^[25]^.

### Alternative treatment options

Oral minoxidil is an additional treatment that can also be used as an effective treatment in male and female patients of AGA^[26]^. The side effects of Oral minoxidil were tolerable and were seen in 30% of patients part of the study^[27]^. A prospective study also showed that when increasing the dose of minoxidil to once-daily 5 mg, the improvement was 100% at weeks 12 and 24^[28]^. The side effects of oral minoxidil included increased heart rate, hypertrichosis, weight gain, hirsutism, and lower extremity edema as well, which makes the drug less favorable as compared to topical minoxidil, although the effects are dose-dependent and reversible^[28]^.

Spironolactone is another available treatment which can potentially assist in AGA. The mechanism of action of spironolactone reduces testosterone production by inhibiting 17a-hydroxylase in the adrenal gland^[29]^. A clinical trial by Sinclair *et al* showed that using 200 mg in 80 participants of the study 44% showed signs of hair growth^[30]^. Furthermore, in a retrospective study of 166 patients who suffered from hair loss, 70% noted an improvement in their disease with the use of spironolactone^[31]^.

Low-level laser therapy (LLLT) is another treatment modality that has been observed to have positive changes in patients with hair loss. Although the exact mechanism by which LLLT works, it is believed that red light absorption by cytochrome C oxidase ultimately leads to photo dissection of nitric oxide in the mitochondria and subsequent ATP production and transcription factor induction^[32]^. In a study with LLLT and placebo, over the course of 16 weeks and alternate-day therapy, a 37% increase in terminal hairs was seen in the treatment group^[33]^.

### New treatment options

Clascoterone is a topical antiandrogen agent that resembles DHT and spironolactone, and its mechanism is believed to antagonize AR on dermal papillae and additionally inhibit DHT’s effect on hair miniaturization^[34]^. In a study conducted over 6 months, patients who received clascoterone bidaily doses showed an improvement in hair loss as compared to those who were prescribed a placebo^[35]^.

JAK inhibitors have been found to use a positive feedback loop to induce signaling in T cells, eventually disrupting the autoimmune attack by IL-15 seen in alopecia areata, where JAK has been considered an effective therapy. This mechanism allows for hair follicles to reenter the anagen phase and would lead to hair growth; however, in the study conducted by Yale *et al*, male patients treated for alopecia areata when given JAK inhibitors developed hair growth in the same pattern as seen in AGA^[36]^.

Latanoprost is a prostaglandin analog which was previously used only for Glaucoma, but its side effect of hypertrichosis enabled it to be considered as a suitable alternative for patients with AGA. In a double blind placebo-controlled study, 16 men with mild AGA were given latanoprost, and the results noted an increase in hair density; however, the limitation in the study was that all patients only suffered from mild forms of AGA^[37]^.

### Need for novel therapeutic approaches

Despite the numerous available treatments for AGA, most therapies for AGA are not approved or have shown little effect in the long run. Current commonly used therapies, such as minoxidil and finasteride, are approved; however, they have multiple adverse effects^[9]^. With treatments such as Hair transplant, the short-term effective results are outweighed by the long-term impacts of the chronic action of DHT in miniaturization of nontransplanted hairs. Additionally, local hormonal action, if recurrently used, can impair regular follicular processes^[9]^.

Novel therapeutic approaches also theorize and experiment with the use of Retinoids in treating AGA. Retinoids are believed to play a role in the treatment of AGA and may show revised results in patients suffering from AGA. The mechanism by which retinoids work directly targets the actions of the genetic pathway, which also plays a role in hair follicle development and AGA. The following section delves into the mechanism of RAand its interaction with genetic pathways involved in AGA, and thus can lead to an alternative treatment for AGA.

## Retinoids and androgenetic alopecia

Recent studies and research have shown the positive effect that topical retinoids have had on AGA. In studies conducted by Shahpar *et al*, we saw that retinoids had a positive and effective treatment on patients suffering from alopecia. In addition to its primary action, retinoids have a supporting effect with drugs like minoxidil in treating patients with alopecia^[38]^. While minoxidil focuses on the anagen phase where hair growth occurs and eventually promotes new hair growth in AGA, Retinol aids by improving the scalp conditions, eventually encouraging hair growth^[39]^.

Several retinoids are commonly used as treatments for various illnesses, including AGA. Categorizing retinoids depends on their potency, irritability and effectivity on multiple skin types and can be classified as RA(tretinoin) which is the most readily active form, retinaldehyde (retinal) which requires one conversion for activation, and stands as the highest form of non prescription available retinoid, retinol a slightly unstable photosensitive form, and retinyl esters the weakest form of retinoids available. During the course of this study, we will be focusing on RA(tretinoin) and its ability to stimulate hair follicle receptors as the main method of treatment used in AGA^[40]^.

### Mechanism of action

RA is a vitamin A metabolite that is produced from Retinol in two steps. Studies by Ghyselinck and Duester showed that RA is synthesized first through retinol dehydrogenase-10, producing retinaldehyde, and simultaneously, the counter-conversion of retinaldehyde to retinol takes place to maintain a balance of RA. Conversion of retinaldehyde through the next steps requires multiple enzymes (which have been identified through mouse and zebrafish studies), such as ALDH1A1 (RALDH1), ALDH1A2 (RALDH2). Although the RA synthesis process is irreversible, it is rapidly degraded by P450 enzymes, thus rendering a very short half-life to RA^[40]^.

RA functions as a ligand for nuclear retinoic acid receptors (RAR), which ultimately bind retinoid X receptors (RXR) as heterodimer complexes, as expressed by Ghyselinck and Duester. This binding occurs at a DNA sequence that is recognized as the RA response element (RARE). This binding allows RARE to recruit either coactivators or corepressors, which subsequently alter gene expression^[40]^.

In the treatment for AGA, Kim *et al* expressed that toll like receptor 3 (TLR3) helps in dsRNA sensing, which ultimately results in RA synthesis and the promotion of regeneration of wound-induced *de novo* hair follicles^[41]^. It was also appreciated by Wen *et al* that RA is the messenger that mediates the interaction between hair follicle stem cells (HFSCs) and melanocyte stem cells. In the HFSC pathway, the prominent signaling mechanism is the Wnt/β-catenin pathway, which activates downstream genes, accumulates in the nuclei, and binds to TCF/LEF, thus in turn activating gene expression. HFSC can fail to differentiate whether either the deletion of β-catenin or Wnt inhibition by DKK1 occurs. There is also evidence of association between the Wnt/β-catenin and RA signaling in the pathogenesis of human disease^[42]^.

On the other hand, we also have evidence of the counteraction androgens have on dermal papillae and the sensitivity regulation that occurs with 5α-reductase, AR, and AR coactivators. Additionally, recent reports show that there is some association between androgens and Wnt/β-catenin pathways. There was also evidence that showed that DHT in the Androgen pathway had inhibitory effects on TCF/LEF activity in the dermal papillae in AGA^[43]^.

**Figure 1.**
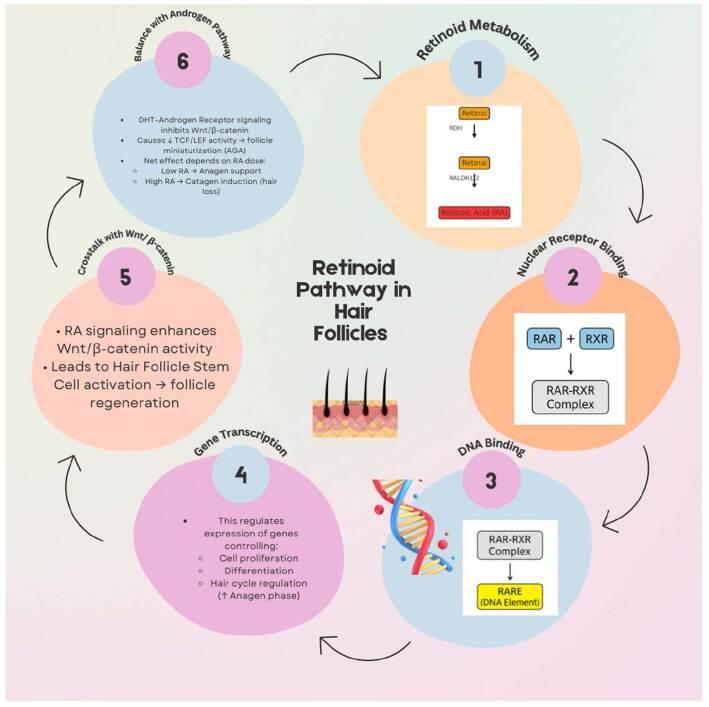
Retinoid pathway in hair follicles. Retinol undergoes enzymatic conversion to retinoic acid (RA), which binds nuclear receptors (RAR/RXR) to form a complex. This complex attaches to RA response elements (RARE) on DNA, initiating transcription of genes regulating cell proliferation, differentiation, and hair cycle progression. RA signaling also interacts with Wnt/β-catenin pathways to activate hair follicle stem cells and promote follicle regeneration, while balancing with androgen receptor activity that may otherwise drive miniaturization in androgenetic alopecia.

The mechanistic pathway of retinoids in hair follicle regulation involves sequential metabolism, nuclear receptor binding, DNA interaction, and crosstalk with Wnt/β-catenin and androgen pathways (Fig. 1).

### Preclinical evidence

Multiple preclinical in vitro studies that have been carried out show that retinol has been able to significantly improve scalp skin condition, and when combined with minoxidil we see that the prolongation of the anagen phase that occurs greatly promotes hair growth. In studies conducted by Kwon *et al*, it was seen that All-trans RA(ATRA) had a positively supportive effect, and ATRA combined with minoxidil enhanced hair growth significantly as compared to Minoxidil alone, suggesting the key role retinoids can play^[44]^.

The actions of retinoids in treatment therapy have been used for years in tackling alopecia and have had a positive impact on alopecia areata and cicatricial alopecia. Studies conducted by Holler and Cotsarelis showed that retinoid therapy was effective in stimulating gene upregulation. The underlying mechanism of action through which retinoids act on hair cells in subjects of alopecia is similar to the proposed mechanism seen in AGA. It is thus believed that clinical studies of the effects of retinoids and AGA would show a positive impact and provide grounds for further research into this treatment modality^[45]^.

### Clinical trials

Multiple *in vivo* studies also showed the positive effects that were speculated with ATRA. Studies conducted by Sharma *et al* showed that when combined with minoxidil showed a positive impact, where almost half of the subjects who initially did not report a response to minoxidil felt a positive response to treatment by the 5th day^[46]^.

Studies reported by Terezakis *et al* showed that two-thirds of subjects showed signs of terminal hair growth and more than half showed some hair regrowth. The study also showed that subjects who had suffered from chronic AGA show signs of regrowth after prolonged use of combined minoxidil tretinoin therapy. This suggested some synergistic effect of minoxidil in combination with retinoids, which should be further investigated^[47]^.

Similarly, in studies conducted by Shin *et al*, it was seen that the effect of minoxidil and a combined minoxidil-retinoic treatment was tested in a double blind study. During the study, the researcher’s parameters included the hair count, terminal hair, the effect of treatments on the anagen phase, and the hair diameter, which was assessed by Macrophtographic image analysis (showcasing an object larger than its size to evaluate it better). The results of this study also showed that the efficacy of the combined once daily therapy was similar to that of the twice daily only minoxidil therapy, suggesting positive effects of the combined treatment; however, further investigation on larger groups over variable demographics would improve our understanding of these results^[48]^.

A recent case series (2025) demonstrated that sequential application of 0.1% tretinoin gel followed by 5% minoxidil over 90 days significantly improved hair density, anagen conversion, and thickness in AGA nonresponders^[49]^. Additionally, a novel Phase I trial (ClinicalTrials.gov Identifier: NCT04913519) is currently underway to assess the safety of a new topical solution in MAGA patients.^[50]^ These encouraging findings underscore the need for well-designed Phase II/III randomized trials of tretinoin–minoxidil combinations to optimize efficacy and minimize irritative drawbacks.

A summary of key clinical trials investigating the use of retinoids, alone or in combination with minoxidil, is provided in Table 1.

**Table 1:** Clinical trials of retinoids in androgenetic alopecia

Study	Design	*N*	Intervention (dose/formulation)	Comparator	Duration	Outcomes	Limitations
Terezakis *et al*, 1986^[47]^	Open-label clinical study	Not specified (~20)	Topical tretinoin (dose not standardized)	None	Prolonged use (months)	Two-thirds subjects showed terminal hair growth; regrowth in chronic AGA cases	Small, uncontrolled, nonstandardized
Shin *et al*, 2007^[48]^	Randomized, double-blind, comparative trial	45 men	5% Minoxidil + 0.01% tretinoin (once daily)	5% minoxidil twice daily	16 weeks	Similar efficacy to minoxidil BID; maintained hair count, thickness	Small sample size; short duration
Sharma *et al*, 2019^[46]^	Prospective clinical trial	56 patients	Minoxidil + topical tretinoin	Minoxidil alone	Variable (response observed by day 5 onward)	Half of the minoxidil nonresponders improved with combination therapy	Limited sample; mechanism inferred
Chandrashekar *et al*, 2025^[49]^	Case series	AGA nonresponders (exact *N* not given in abstract)	0.1% Tretinoin gel + 5% minoxidil (sequential application)	Prior minoxidil nonresponders	90 days	~33% Increase in density, ↑ anagen hairs, ↑ thickness	Case series; short follow-up; uncontrolled
NCT04913519 (ongoing)	Phase I clinical trial	Male AGA patients (safety study)	Novel topical solution (TDM-105 795)	Placebo/control arm for safety	Ongoing	Assessing safety/tolerability only	Early phase; no efficacy data yet

## Risk–benefit analysis

Topical retinoids are increasingly being explored as supportive treatments for AGA, with growing evidence pointing to their potential clinical benefits. Studies over the years have proven that retinoids improve the percutaneous absorption of 2% minoxidil by threefold^[8,51]^. Retinoids increase stratum corneum permeability, thereby enhancing the percutaneous absorption of agents such as minoxidil^[8,51]^. Greater efficacy also results in better adherence to treatment by the patients since this reduces the number of daily applications and results in better compliance with treatment. However, retinoids are associated with certain risks. Risks seen with the use of retinoids present as Telogen Effluvium, Local irritation along with skin-associated side effects, frontal fibrosing alopecia, and alopecia areata^[8]^. The majority of the risks posed by the use of retinoids are reversible when the drug is tapered off or discontinued. Teratogenic side-effects of retinoids also warrant vigilance when used in pregnancy or childbearing age^[52]^. Risks associated with retinoids are manageable, and under the correct professional supervision, they make a viable and effective treatment option.

While the use of retinoids presents with a favorable risk–benefit ratio when used topically, especially in association with other topical agents, assessment of the risk–benefit ratio for the use of retinoids is integral for making informed clinical decisions. Retinoids have proven to improve the symptoms of AGA and the presenting risks that come along with the use of topical retinoids are proven to be reversible and manageable.

A comparative overview of retinoids, minoxidil, and finasteride with respect to their mechanisms, efficacy, limitations, and safety is presented in Table 2.

**Table 2:** Comparative summary of the mechanisms, efficacy, benefits, limitations, adverse effects, and long-term safety of retinoids, minoxidil, and finasteride in the management of androgenetic alopecia

Feature	Retinoids	Minoxidil	Finasteride
Mechanism of action	Regulation of the proliferation/differentiation of keratinocytes; WNT/β-catenin upregulation; potential to enhance other topical agents	Anagen phase prolongation; dilates vessels; modulates potassium channel opening;	5α-Reductase type II inhibitor → ↓DHT at the hair follicle level
Efficacy	Has limited action as monotherapy; can be synergistic with minoxidil; preliminary studies suggest hair density improvements in some smaller trials	40–60% Response; shown to be more effective in younger patient; continuous use is required;	More effective in younger men less than 40 years of age; 80–90% stabilization; best in vertex/mid-scalp.
Onset of effect	Needs more trial evidence; initial studies show a slower onset, taking months.	3–6 months	6–12 months
Benefits	Follicle regeneration may be activated potentially through the WNT pathway; possible normalization of follicular keratinization with increased penetration of the scalp by topicals;	Available widely for topical use; synergistic with retinoids and safe in the long term;	AGA progression is reduced, and stabilization in the long-term; Oral; strong evidence from RCTs.
Limitations	Large RCTs are not readily available; adherence to the drug is limited by irritation; teratogenic	Continuous use is required; decreased effectiveness on the frontal hairline.	Mainly prevents loss and is not effective in women (some postmenopausal effectiveness)
Common adverse effects	Photosensitivity, erythema, irritation, dryness; systemic forms: teratogenic, hepatotoxic, dyslipidemia	Dermatitis (propylene glycol), Irritation of the scalp, itching scalp irritation, itching, dermatitis, hypertrichosis (unwanted hair)	Gynecomastia, Sexual dysfunction (↓libido, ED, ejaculatory disorder); some cases of depression
Comorbidities/risks (from cohort data)	May aggravate eczema/dermatitis; Topical forms not linked directly to systemic disease	Systemic risks are rare; minimal effect on metabolic profile	Use shows increased association with depression, prostate disease, metabolic changes (↑diabetes, vascular disease, obesity prevalence in high-dose cohorts)
Long-term safety	Topical is generally safe, but systemin is not suitable for androgenic alopecia	Continuous use is safe, and adverse effects are reversible	Monitoring is needed in long-term use; concerns for safety due to persisting sexual dysfunction (post-finasteride syndrome).
Evidence base	Preclinical and clinical studies are limited and, although promising, may be inefficient for guidelines	Standard treatment widely used with multiple RCTs; FDA-approved	Strong supportive guidelines, widely used with multiple RCTs; FDA-approved;
Overall role	Potential to adjunct other topical agents and possibly enhance their action in follicular regeneration	First-line topical with long-term adherence for best results	First-line systemic therapy in men with more preventative action as compared to restorative

## Challenges and future directions

While retinoids show a promising potential for the treatment of AGA with minoxidil, their stand-alone efficacy remains unproven so that is why there is a need to do more research to determine their independent effects, optimal formulations, and long-term safety. Clinical trials and mechanistic studies are essential to establish whether they work alone or merely enhance treatments like minoxidil and finasteride^[46]^. Other than this one of the primary concerns is the adverse effect profile that it carries. Retinoids are known to cause hair loss and thinning, particularly the systemic ones like isotretinoin, where they act as catagen to induce premature hair follicle regression by augmenting the transformation of beta-2, a growth factor embedded in the dermal papilla^[53]^. Moreover, this retinoid-induced hair loss can manifest as telogen effluvium, where hair sheds in clumps^[54]^. In FAGA, where diffuse thinning is already prevalent, retinoid-induced telogen effluvium may be especially distressing. The mechanism likely involves premature catagen induction through upregulation of TGF-β2 within the dermal papilla, accelerating follicular regression^[53]^. This highlights the importance of sex-specific safety evaluations when retinoids are tested in AGA populations. Dermatitis has also been reported in individuals with sensitive scalps, manifesting as itching, redness, and peeling after retinoid use with its careless overdosage^[55]^. Higher concentrations or systemic absorption of retinoids can lead to severe effects like teratogenicity and hepatotoxicity^[56,57]^. This raises concern for their safe widespread application. Although tretinoin (a retinoid) has been proven efficacious with the dosage of 0.01% with 5% minoxidil for AGA, there has not been a proper guideline on the dosage to be used with a specific delivery method; therefore, optimizing the formulation and delivery methods of retinoid-based therapies is crucial^[48]^.

One promising avenue for future research is the development of novel retinoid analogs. By modifying the chemical structure of existing retinoids, researchers may be able to enhance their therapeutic effects while minimizing side effects. For instance, selective RAreceptor modulators are being explored in other dermatological applications and could be adapted for AGA treatment. These compounds could provide targeted activation of beneficial pathways without triggering the full range of adverse effects associated with traditional retinoids^[58]^.

Another key area for future exploration is the use of advanced drug delivery systems. Topical retinoids are often associated with poor penetration and systemic absorption issues^[59]^. Nanotechnology-based delivery systems, such as liposomes and nanoparticles, could allow for controlled release and targeted delivery to the hair follicle, reducing systemic side effects^[60]^. Microneedling, already used in dermatology to enhance topical drug absorption, could further improve the efficacy of retinoid treatments for AGA^[61]^.

Finally, more extensive clinical trials are needed to establish the efficacy and safety of retinoids in treating AGA. Current research is limited, with most data derived from preclinical studies and small-scale clinical observations. Future studies should focus on long-term outcomes, optimal dosing strategies, and patient satisfaction to determine whether retinoids can become a viable addition to the AGA treatment landscape.

## Conclusion

The emerging role of retinoids as a therapeutic option for AGA highlights their capacity to influence hair follicle biology through regulation of cellular proliferation, differentiation, and signaling pathways. By modulating mechanisms such as Wnt/β-catenin activation and follicular stem cell dynamics, retinoids may extend the anagen phase and counteract follicular miniaturization. Preclinical and early clinical findings also suggest that retinoids may enhance responses to established therapies like minoxidil, underscoring their relevance in combination regimens.

Despite these promising insights, the therapeutic application of retinoids in AGA remains in its infancy. Current evidence is limited to small studies, short durations, and heterogeneous protocols, leaving key questions about optimal formulations, long-term safety, and independent efficacy unresolved. Adverse effects, including retinoid-induced telogen effluvium and irritant dermatitis, further highlight the need for careful dose optimization and patient selection.

Retinoids hold potential as adjuncts or future alternatives in AGA therapy, but current evidence remains preliminary. Their integration into treatment protocols requires validation in large, long-term randomized trials.

## Data Availability

No data is available.

## Research registration unique identifying number (UIN)

Once registered, you will need to submit your assigned unique identifying number (UIN) from your registration body as a mandatory part of your submission.
